# Digital panoramic radiography and CBCT as auxiliary tools for detection of low bone mineral density in post-menopausal women: a cross-sectional study

**DOI:** 10.1186/s12880-023-01046-x

**Published:** 2023-06-12

**Authors:** Mehrdad Abdinian, Mina Milaei, Parisa Soltani

**Affiliations:** 1 Department of Oral and Maxillofacial Radiology, Dental Implants Research Center, Dental Research Institute, School of Dentistry, Isfahan University of Medical Sciences, Hezar-Jarib Ave, P.O. 8174673461, Isfahan, Iran; 2 Student Research Committee, School of Dentistry, Isfahan University of Medical Sciences, Isfahan, Iran; 3 Department of Neurosciences, Reproductive and Odontostomatological Sciences, University of Naples “Federico II”, Naples, Italy

**Keywords:** Cone beam computed tomography, Panoramic radiography, Bone mineral density, Dual energy x-ray absorptiometry, Postmenopausal

## Abstract

**Background:**

Osteoporosis is a chronic, multifactorial skeletal disease that occurs especially in women following a decrease in estrogen levels and decrease in bone mineral density. The aim of this study was to evaluate the relationship between qualitative and quantitative indexes in panoramic radiographs and quantitative indexes in CBCT images with femoral and vertebral BMD in postmenopausal women.

**Methods:**

This comparative cross-sectional study was performed on postmenopausal women aging 40 to 80 years attending for obtaining either panoramic radiograph or mandibular CBCT scan. Dual energy X-ray absorptiometry (DEXA) was performed from the femur and lumbar vertebra. Quantitative parameters of mental index (MI), panoramic mandibular index (PMI), antegonial index (AI) as well as qualitative parameters of mandibular cortical index (MCI) and trabecular bone pattern (TP) were evaluated in panoramic radiographs. Quantitative parameters computed tomography mandibular index (CTMI), computed tomography index (inferior) [CTI(I)] and computed tomography index (superior) [CTI(S)] were analyzed in CBCT images. Kolmogorov-Smirnov tests and Pearson correlation coefficient were used (α = 0.05).

**Results:**

In individuals with panoramic radiography, statistically significant correlations were observed between MI with vertebral and femoral T-score, AI with vertebral and femoral T-score (except for the right AI with femoral T-score), and TP with vertebral and femoral T-score (p < 0.05). In the group with CBCT scans, the correlations between CTMI with vertebral and femoral T-score, CTI(I) with vertebral and femoral T-score, and CTI(S) with vertebral and femoral T-score were statistically significant (p < 0.05).

**Conclusions:**

in CBCT images, quantitative indexes of CTMI, CTI(I), and CTI(S), and in panoramic images, quantitative indexes of MI and AI and qualitative index of TP can be used to predict the possibility of osteoporosis in postmenopausal women.

## Background

Osteoporosis is a chronic, multifactorial disease which occurs as a result of a decrease in the mineral density of the bone. It often affects older individuals and gradually results in susceptibility to bone fractures [1]. Postmenopausal women are the most at-risk population for osteoporosis as a result of the decrease in estrogen production [2]. Osteoporosis has an insidious nature and can go unnoticed until advanced manifestations such as skeletal fractures occur. Timely diagnosis of osteoporosis is of utmost importance as it can significantly improve the quality of life of affected individuals and reduce the financial burden of therapeutic approaches [3, 4].

Dual energy X-ray absorptiometry (DEXA) is the gold standard for determination of bone mineral density (BMD). BMD values are usually measured in the femur and lumbar vertebra [5, 6]. According to the classification proposed by World Health Organization, the standard deviation values from normal BMD is considered as the T-score. BMD values equal to or more than − 1 are normal, while osteopenia refers to a T-score between − 2.5 and − 1 and osteoporosis is defined by a T-score equal to or less than − 2.5 [7]. DEXA is a reliable diagnostic approach. However, it has shortcomings such as inability to differentiate between cortical and trabecular bone, limited availability and personnel, and high expenses. Therefore, application of adjunct cost-effective approaches is suggested [8].

Today, more and more individuals in older age are seeking dental services, a lot of them being partially or completely edentulous and in need of dental implant treatments. Panoramic radiography and cone beam computed tomography (CBCT) are among the main radiographic modalities in the preoperative and treatment planning phases of implant treatments [9, 10]. Panoramic radiography provides a screening view of the dentition and edentulous regions, as well as the anatomical landmarks [11]. CBCT images provide a three-dimensional view of the dentoalveolar structures, allowing for evaluation of the quality and quantity of available bone [12, 13]. Application of these modalities for identification of patients with low BMD can be helpful for earlier diagnosis and preventing more complicated consequences of osteoporosis [3, 14, 15].

Several studies have used different features of dental diagnostic images for prediction of individuals with low BMD [4]. Studies have shown that in CBCT images, features such as gray value in different areas of the jaw are associated with femoral and vertebral BMD [16, 17]. Additionally, different mandibular parameters in panoramic images have been shown to be correlated to BMD [18, 19]. However, most of the studies on panoramic imaging focus on qualitative indexes. To the authors’ knowledge, no previous study has been performed on both qualitative and quantitative indexes in CBCT and panoramic radiographs in a group of postmenopausal individuals. Performing the analysis on the same group of individuals can further establish the role of dental radiographic modalities in detection of low BMD. Therefore, the present study aimed to evaluate the relationship between qualitative and quantitative indexes in panoramic radiographs and quantitative indexes in CBCT images with femoral and vertebral BMD in postmenopausal women.

## Methods

This study was ethically approved by Research Ethics Committee at Isfahan University of Medical Sciences (#IR.MUI.RESEARCH.REC.1400.030, approval date: 19/4/2021). The study protocol conformed to the Declaration of Helsinki and informed consent form was signed by all study participants.

This comparative cross-sectional study was performed on postmenopausal women aging 40 to 80 years attending the Department of Oral and Maxillofacial Radiology at Isfahan School of Dentistry for obtaining either panoramic radiograph or mandibular CBCT scan from April to August 2021. The sample size was determined considering a power of 80% and α = 0.05 as 40 individuals in each group.

Inclusion criteria were lack of systemic disorders including hyperparathyroidism, Paget’s disease, osteomalacia, osteogenesis imperfecta, diabetes, and metabolic and renal diseases, lack of uptake of drugs affecting bone metabolism, hormonal therapy, and calcium and vitamin D supplements for the last 6 months, surgical procedures on ovaries, smoking, drug abuse, and history or radiographic evidence of head and neck trauma. Patients who were unwilling to participate in the study, those with DEXA examination in the last 12 months, and individuals whose CBCT or panoramic images did not have appropriate quality were excluded from the study.

DEXA was performed from the femur and lumbar vertebra.

**Fig. 1:**
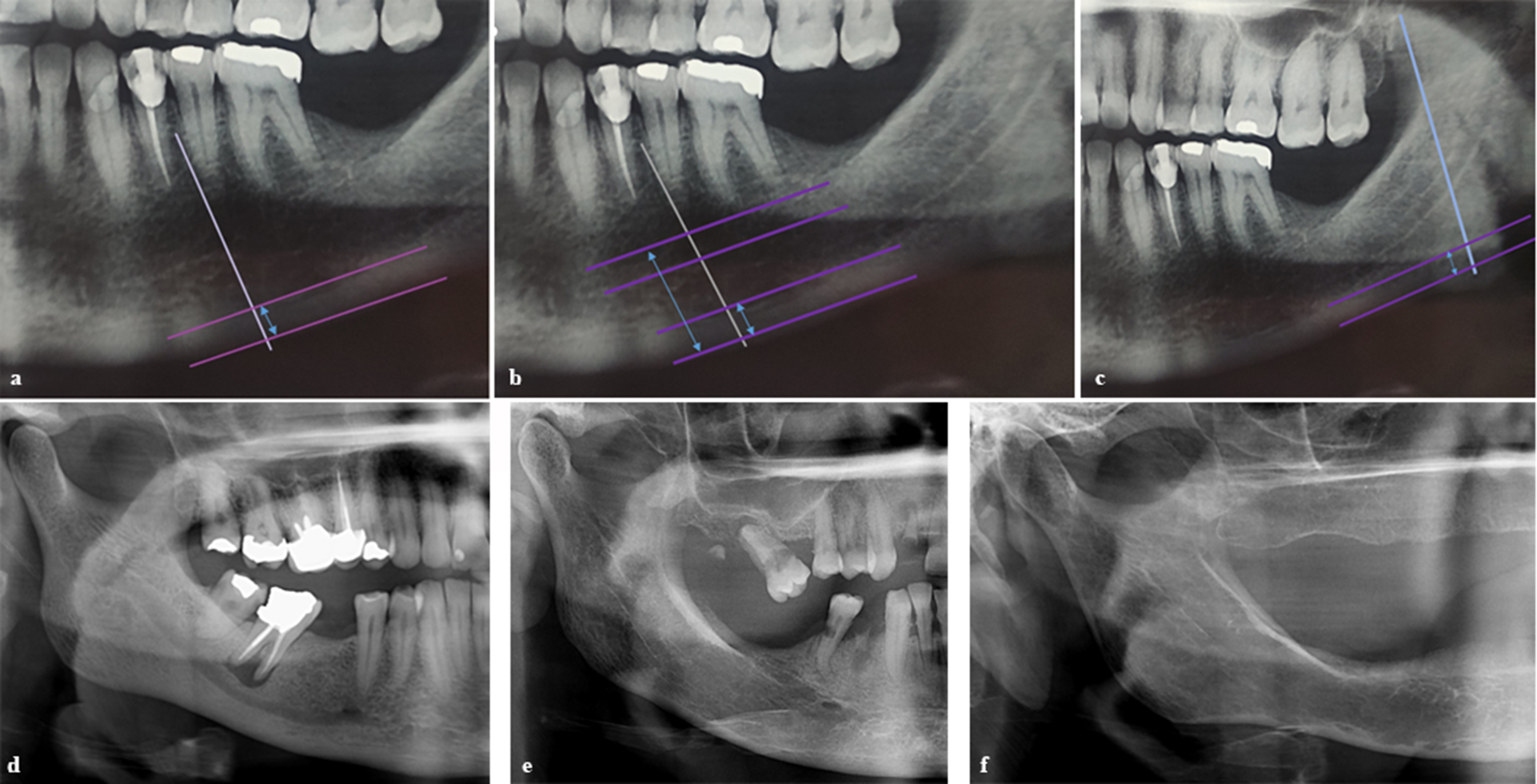
Analyzed indexes in panoramic radiographs: (**a**) mental index, (**b**) panoramic mandibular index, (**c**) antegonial index, (**d**) mandibular cortical index class 1 and dense trabecular pattern, (**e**) mandibular cortical index class 2 and heterogenous trabecular pattern, and (**f**) mandibular cortical index class 3 and sparse trabecular pattern

**Fig. 2:**
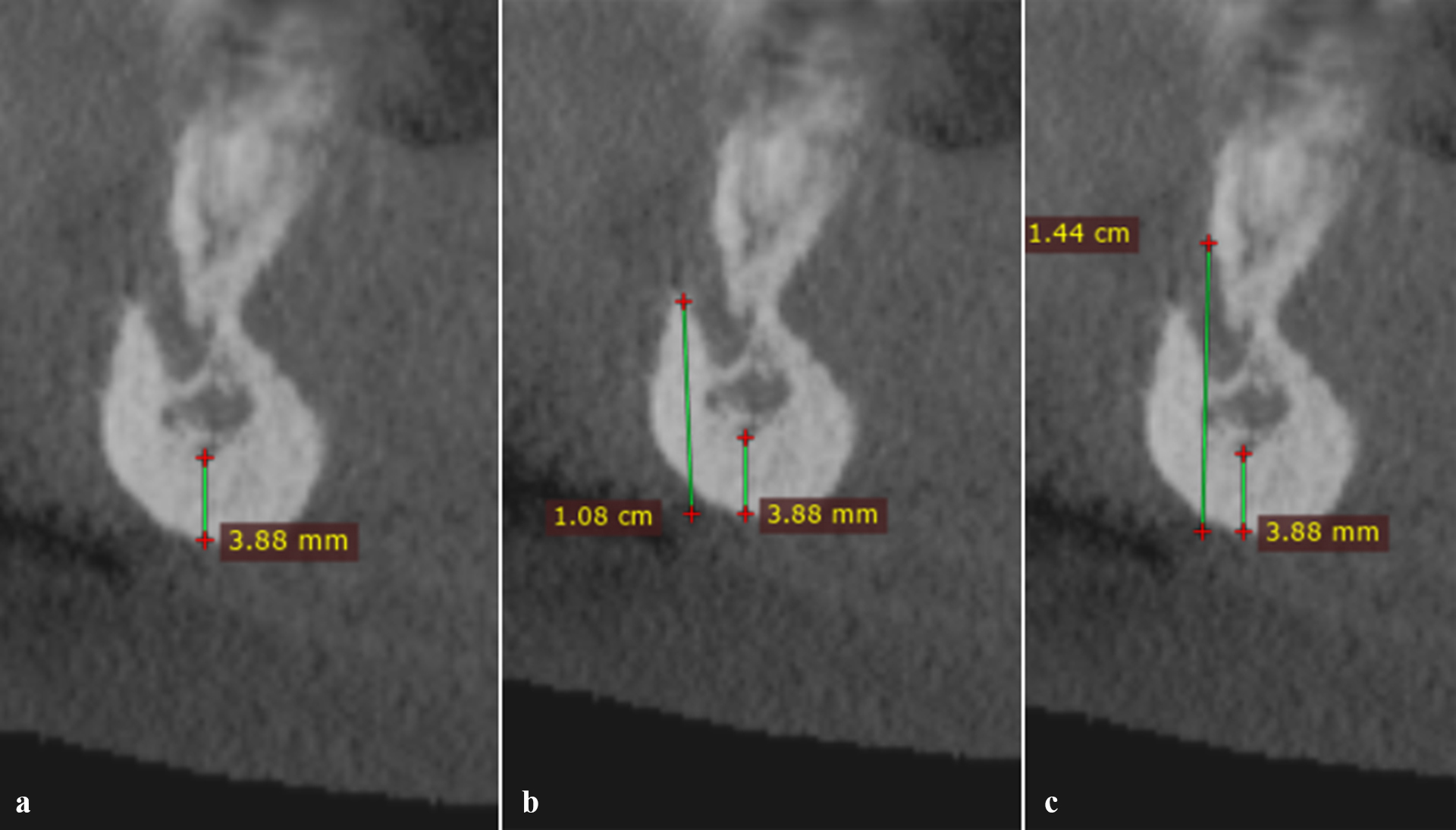
Analyzed indexes in cone beam computed tomographic images: (**a**) computed tomography mental index, (**b**) computed tomography index (inferior), and (**c**) computed tomography index (superior)

Panoramic radiographs were obtained using Promax radiographic unit (Planmeca, Helsinki, Finland) with the patients’ Frankfurt plane parallel with and midsagittal plane perpendicular to the horizontal plane. Exposure parameters optimized for each individual. The images were analyzed in Romexis software (Planmeca, Helsinki, Finland). Mental index (MI), panoramic mandibular index (PMI), and antegonial index (AI) were the quantitative parameters measured in panoramic radiographs (Fig. 1, a-c). Additionally, qualitative parameters including mandibular cortical index (MCI) and trabecular bone pattern (TP) were also analyzed in panoramic radiographs (Fig. 1, d-f). CBCT images were obtained by Galileos scanner (Sirona, Bensheim, Germany) with the patients’ Frankfurt plane parallel with and midsagittal plane perpendicular to the horizontal plane. A voxel size of 0.125 mm and optimized exposure parameters for each individual were selected. The DICOM file of the scans were extracted and imported into Radiant software (Medixant, Poznań, Poland). Quantitative parameters measured in CBCT images were computed tomography mandibular index (CTMI), computed tomography index (inferior) [CTI(I)] and computed tomography index (superior) [CTI(S)] (Fig. 2a-c). The analyzed parameters are defined in Table 1.

**Table 1:** Indexes analyzed in panoramic radiographs and CBCT images

Index	Definition
Mental index	The inferior cortical width of the mandible in the mental foramen region in panoramic radiographs
Panoramic mandibular index	The ratio of the thickness of the mandibular cortex to the distance between the mental foremen and the inferior mandibular cortex in panoramic radiographs
Antegonial index	The cortical width in the region anterior to the gonial at a point identified by extending a line of best fit on the anterior border of mandible in panoramic radiographs
Mandibular cortical index	Mandibular cortical shape distal to the mental foramen in panoramic radiographs: Class 1 (normal cortex): the endosteal margin of the cortex is even and sharp on both sides. Class 2 (mildly to moderately eroded cortex): the endosteal margin has semilunar defects (lacunar resorption) or appears to form cortical residues on one or both sides. Class 3 (severely eroded cortex): the cortical residues are clearly porous on one or both sides.
Trabecular pattern	Pattern of bone trabeculation in the alveolar process area distal to the mental foramen: Dense: Uniform dense bone trabeculae Heterogenous: Non-uniform bone trabeculae Sparse: Uniform porous bone trabecula
Computed tomography mandibular index	The inferior cortical width of the mandible in the mental foramen region in coronal CBCT images
Computed tomography index (inferior)	The ratio of the inferior cortical width to the distance from the inferior margin of the mental foramen to the inferior border of the mandible in coronal CBCT images
Computed tomography index (superior)	The ratio of the inferior cortical width to the distance from the superior margin of the mental foramen to the inferior border of the mandible in coronal CBCT images

All qualitative measurements were performed by two independent oral and maxillofacial radiologists (with 11 and 5 years of experience) after a calibration session on 7 panoramic radiographs and 7 CBCT images unrelated to the study. In case of disagreement, a third oral and maxillofacial radiologist was consulted. Quantitative measurements were performed by a senior dental student who was trained by the radiologists using 7 radiographic images unrelated to the study. Additionally, 20% of the images were analyzed again after two weeks by the observers.

Intraoberver and interobserver agreements were calculated using intraclass correlation coefficient and Cohen’s kappa. Kolmogorov-Smirnov test and Pearson correlation coefficient were used for statistical analysis. Statistical analysis was performed using Statistical Package for the Social Sciences (SPSS, v 27, IBM, NY, USA).

## Results

A total of 80 postmenopausal women enrolled in the present study. Forty of them had panoramic radiographs and the other forty had CBCT scans. The participants were between 40 and 77 years old (57.55 ± 9.84). All the variables had normal distribution and therefore parametric tests were used for statistical analysis. Interobserver and intraobserver agreements were high (κ 0.903–0.920, p < 0.001 and ICC 0.903–0.950, p < 0.001).

In individuals with panoramic radiography, statistically significant correlations were observed between MI with vertebral and femoral T-score, AI with vertebral and femoral T-score (except for the right AI with femoral T-score), and TP with vertebral and femoral T-score (P < 0.05, Table 2). Other panoramic indexes were not significantly related to T-score (P > 0.05). The most significant correlation was seen for AI and vertebral T-score (r = 0.532, P = 0.001 and r = 0.394, P = 0.046, respectively for the right and left side). In the group with CBCT scans, the correlations between CTMI with vertebral and femoral T-score, CTI(I) with vertebral and femoral T-score, and CTI(S) with vertebral and femoral T-score were statistically significant (P < 0.05, Table 3). No significant correlation was observed between other CBCT indexes and T-score (P > 0.05). The most significant correlation was seen for CTMI and vertebral T-score (r = 0.520, P = 0.001 and r = 0.367, P = 0.020, respectively for the right and left side).

**Table 2:** The relationship between different indexes in panoramic radiographs and vertebral and femoral T-scores

		Vertebral T-score		Femoral T-score	
		r	P-value	r	P-value
MI	Right	0.350	0.034	0.365	0.026
	Left	0.367	0.025	0.410	0.012
PMI	Right	0.269	0.107	0.125	0.461
	Left	0.244	0.146	0.176	0.298
AI	Right	0.532	0.001	0.460	0.004
	Left	0.394	0.046	0.289	0.083
MCI	Right	-0.241	0.152	-0.148	0.380
	Left	-0.238	0.157	-0.120	0.480
TP	Right	-0.404	0.013	-0.507	0.001
	Left	-0.369	0.025	-0.485	0.002

MI: mental index

PMI: panoramic mandibular index

AI: antegonial index

MCI: mandibular cortical index

TP: trabecular pattern

**Table 3:** The relationship between different indexes in CBCT images and vertebral and femoral T-scores

		Vertebral T-score		Femoral T-score	
		r	P-value	r	P-value
CTMI	Right	0.520	0.001	0.368	0.014
	Left	0.367	0.020	0.354	0.025
CTI(I)	Right	0.481	0.002	0.358	0.023
	Left	0.354	0.025	0.358	0.023
CTI(S)	Right	0.433	0.005	0.350	0.027
	Left	0.338	0.033	0.352	0.026

CTMI: computed tomography mandibular index

CTI(I): computed tomography index (inferior)

CTI(S): computed tomography index (superior)

## Discussion

Based on the findings, different parameters in panoramic radiographs and mandibular CBCT scans are associated with vertebral and femoral BMD. These finding highlights the potential of routine dental radiographs for identifying patients at-risk for osteoporosis.

In the present study, a weak but statistically significant correlation was observed between MI, i.e., mandibular cortical width in the region of mental foramen, with the vertebral and femoral T-score. Similar to our findings, in a study by Gaur et al., the association between MI and lumbar and femoral BMD [20]. Additionally, Valerio et al. in their study investigated the relationship between the mandibular cortical width in different locations and lumbar T-score. They found that in addition to MI, mandibular cortical width in 1-cm, 2-cm, and 3-cm posterior to the mental foramen is also significantly different between postmenopausal women with normal BMD and those with osteopenia or osteoporosis. The authors discussed that since finding the exact location of the mental foramen may be challenging in some panoramic radiographs, this additional information is useful for application of panoramic radiographs in screening postmenopausal women [19]. In another study by Tofangchiha et al., MI had a high diagnostic accuracy for detecting osteoporotic patients [21]. In addition, Navabi et al. in their study of 50 postmenopausal women showed that MI is appropriate for diagnosis of individuals with osteoporosis [18]. Ezoddini et al. evaluated 60 panoramic radiographs stated that a threshold of 2.8 mm for mandibular cortical width in the angle region can be considered in Iranian population for suggesting low BMD [22]. Hekmatian et al. additionally reported a significant relationship between MI and BMD in postmenopausal women [23]. Kaveyani et al. evaluated several indexes in panoramic radiograph of 140 postmenopausal women and reported that a 1-mm decrease in MI, increases the chance of osteoporosis and osteopenia by 40% [24]. Additionally, in their systematic review and meta-analysis, Calciolari et al. reported that MI has a better specificity in detection of individuals with low BMD compared with other mandibular indexes in panoramic radiographs. They mentioned that patients with a MI of more than 4 mm had a normal BMD in 90% of the cases [25]. Devlin et al. in the OSTEODENT project reported that patients with a MI of less than 3 mm should be referred for further osteoporosis investigation [26]. In contrast to the above-mentioned studies, Akshita et al. showed that MI does not have a significant relationship with BMD [27]. The difference can be attributed to populational differences as well as difficulty in precise localization of the mental foramen. Additionally, in the study performed by Soltani et al., MI was not significantly different between pre- and post-menopausal women [28].

Regarding PMI, it has been noted that in spite of alveolar ridge resorption superior to the mental foramen, the distance between the mandibular inferior border and the mental foramen remains constant. Additionally, PMI is a ratio attempting to correct for the magnification in radiographic images. In the present study, PMI, i.e., mandibular cortical width in the mental region divided by the distance between the mental foramen and the inferior mandibular border, was not significantly related to vertebral or femoral BMD. Similar to our findings, Akshita et al. did not find any significant correlation between any of the quantitative indexes in panoramic images and BMD scores [27]. However, Gaur et al. revealed a correlation between PMI and BMD obtained by DEXA [20]. Limited repeatability in measurement of PMI, observer differences, and populational variability can be accounted for the differences observed in the findings of different studies.

Based on our findings, a significant relationship existed between AI and femoral and vertebral BMD. This result is consistent with the findings of Tofangchiha et al. [21] Whereas, Gaur et al. stated that AI is not an appropriate index for screening purposes, as determination of the line tangential to the anterior border of ascending ramus is difficult [20].

In our study, the relationship between mandibular cortical morphology distal to the mental foramen and femoral or vertebral BMD was not significant. Navabi et al. also did not find a significant relationship between these variables [18]. In addition, Soltani et al. reported that MCI is not significantly different between pre- and post-menopausal women [28]. In the meta-analysis by Calciolari et al., mandibular cortical erosion gave an estimated specificity of 0.562 and 0.643 for detecting osteopenia and osteoporosis, respectively. The overall sensitivity of mandibular cortical erosion for detection of osteopenia and osteoporosis was 0.789 and 0.806, respectively [25]. Pallagati et al. argued that mean accuracy value of MCI was 58.0%, 63.3%, and 64.0% for detection of individuals with normal BMD, osteopenia, and osteoporosis, respectively [29]. The variability of the findings of different studies can be due to observer differences, different panoramic unit equipment, different populations, and technique-sensitivity of panoramic radiographs.

In this study, trabecular pattern was significantly associated to femoral and vertebral BMD. Similarly, White et al. showed that trabecular features of the mandible were significantly different among three BMD groups of normal, osteopenic, and osteoporotic [30]. In addition, Taguchi et al. reported that mandibular trabecular bone pattern might be useful for identification of females at-risk of osteoporotic fractures [31].

Based on the findings, a significant relationship existed between CTMI in CBCT images with femoral and vertebral T-score. Mostafa et al. in their study concluded that in CBCT images, the mandibular cortical width is significantly lower in osteoporotic patients compared to those with normal BMD [32]. Güngör et al. in another study revealed that in osteoporotic individuals the mandibular radiomorphometric indexes, including CTMI was significantly lower than the normal BMD group [33]. In contrast with these findings, Koh and Kim failed to find a significant relationship between the linear measurement of CTMI and BMD in postmenopausal women [34]. The difference in the findings can be attributed to small sample size of the study by Koh and Kim.

In this study, a significant relationship existed between CTI(I) and CTI(S) with femoral and vertebral T-score. Similar to our findings, Koh and Kim observed a significant difference in CTI(I) and CTI(S) between normal and osteoporotic women [34]. Brasileiro et also reported that CTI(I) and CTI(S) are significantly lower in osteoporotic women compared to those with normal BMD or osteopenia. However, CTI(I) was not significantly different between normal and osteopenia groups [35].

One of the limitations of this study was the lack of presence of both imaging methods of panoramic radiograph and CBCT for all of the study participants. Additionally, in further studies, using reformatted panoramic images obtained by CBCT scans can be considered for direct comparison of panoramic and CBCT findings. For instance, de Castro et al. developed a new composite osteoporosis index called 3D MOI from two quantitative measures evaluating MCW on panoramic reconstruction images and on cross-sectional images and one qualitative measure assessing cortical bone quality [36]. This approach as well as combining the most useful indexes to yield a more efficient composite index can lead to better diagnosis of osteoporosis and osteopenia based on routinely obtained dental radiographic examinations.

## Conclusion

Based on the findings of this study, in CBCT images, quantitative indexes of CTMI, CTI(I), and CTI(S), and in panoramic images, quantitative indexes of MI and AI and qualitative index of TP can be used to predict the possibility of osteoporosis in postmenopausal women.

## Data Availability

The data supporting this manuscript are available from the corresponding author upon reasonable request.
